# Pharmaceutical pollution disrupts the behavior and predator-prey interactions of two widespread aquatic insects

**DOI:** 10.1016/j.isci.2022.105672

**Published:** 2022-11-25

**Authors:** Aneesh P.H. Bose, Erin S. McCallum, Mladen Avramović, Michael G. Bertram, Eva-Lotta Blom, Daniel Cerveny, Sara N. Grønlund, Johan Leander, Petter Lundberg, Jake M. Martin, Marcus Michelangeli, Lo Persson, Tomas Brodin

**Affiliations:** 1 Department of Wildlife, Fish, and Environmental Studies, Swedish University of Agricultural Sciences, Skogsmarksgränd, Umeå, Västerbotten 907 36, Sweden; 2 University of South Bohemia in Ceske Budejovice, Faculty of Fisheries and Protection of Waters, South Bohemian Research Center of Aquaculture and Biodiversity of Hydrocenoses, Zatisi 728/II, Vodnany, Czech Republic; 3 Department of Science and Environment, Roskilde University, Universitetsvej 1, 4000 Roskilde, Denmark

**Keywords:** Natural sciences, Earth sciences, Environmental science, Environmental chemistry, Ecology

## Abstract

Pharmaceutical pollution represents a rapidly growing threat to ecosystems worldwide. Drugs are now commonly detected in the tissues of wildlife and have the potential to alter the natural expression of behavior, though relatively little is known about how pharmaceuticals impact predator-prey interactions. We conducted parallel laboratory experiments using larval odonates (dragonfly and damselfly nymphs) to investigate the effects of exposure to two pharmaceuticals, cetirizine and citalopram, and their mixture on the outcomes of predator-prey interactions. We found that exposure to both compounds elevated dragonfly activity and impacted their predation success and efficiency in complex ways. While exposure to citalopram reduced predation efficiency, exposure to cetirizine showed varied effects, with predation success being enhanced in some contexts but impaired in others. Our findings underscore the importance of evaluating pharmaceutical effects under multiple contexts and indicate that these compounds can affect predator-prey outcomes at sublethal concentrations.

## Highlights

• Pharmaceutical pollution is a rapidly growing threat to ecosystems worldwide
• Little is known about how pharmaceuticals affect predator-prey interactions
• We exposed dragonfly and damselfly nymphs to cetirizine, citalopram, and their mix
• Both pharmaceuticals affected success and efficiency of the predators

## Graphical abstract

**Figure:**
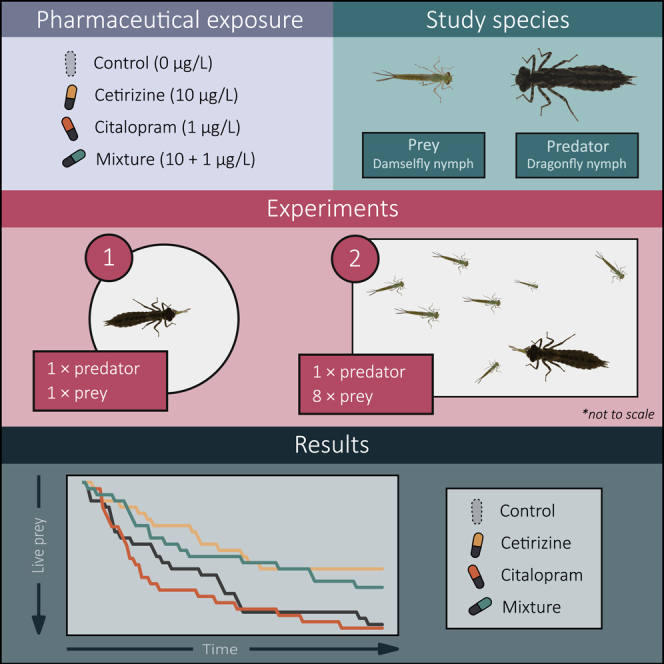

## Introduction

Chemical pollution is altering the functioning of ecosystems globally. Ecosystems are being confronted not only with so-called “legacy contaminants” such as dichlorodiphenyltrichloroethane (DDT), polychlorinated biphenyls (PCBs), and metals (e.g., lead and mercury) but also with a multitude of emerging contaminants that have received comparatively less research attention.^1^ This is largely due to the rapid proliferation of the global chemicals industry, with chemical production having increased 50-fold since 1950, and being predicted to triple again by 2050 relative to 2010.^2^ The annual production and release of new chemicals are increasing at rates that far surpass the global capacity for chemical assessment and monitoring.^3^ Among the classes of emerging contaminants are pharmaceuticals. These compounds are being released into the environment during their manufacture, use, and disposal, and possess a suite of characteristics that make their presence in the environment concerning.^4^ This includes the fact that pharmaceuticals are specifically designed and/or administered to elicit a biological response and can have effects even at relatively low exposure levels (e.g., the nanogram per liter range). Pharmaceuticals are used around the world and are therefore widely dispersed in the environment.^5^ In a recent global census of pharmaceutical pollution in rivers, it was found that in 258 rivers sampled across 104 countries of all continents, 26% of sites had at least one drug at higher concentrations than what are considered safe for aquatic organisms or that is hazardous because of a potential to select for antibiotic resistance.^6^

Once present in the environment, pharmaceuticals can be taken up into the tissues of wildlife by various routes, such as through the skin and respiratory organs, as well as via food and water ingestion, and from contact with contaminated sediment^7^^,^^8^^,^^9^). Consequently, pharmaceuticals have now been detected in organisms from a wide range of taxa, both vertebrate and invertebrate alike.^9^^,^^10^^,^^11^^,^^12^ This is alarming given that exposure to pharmaceuticals has been linked to adverse effects in wildlife. Exposures to certain pharmaceuticals have been the cause of entire population collapses via direct toxicity^13^ (vultures grazing on diclofenac-treated carcasses received toxic doses) or failed recruitment in subsequent generations^14^ (fathead minnows experienced reproductive failure after multi-year, low-concentration exposure to 17α-ethinylestradiol). Exposure to pharmaceuticals can also produce sublethal effects, including altering development, morphology, physiology, metabolism, and reproductive traits.^15^^,^^16^^,^^17^^,^^18^ A growing body of research is also demonstrating that exposure to pharmaceuticals can affect animal behavior,^19^^,^^20^ which can have fitness consequences for individuals living in contaminated habitats.^21^ For example, European perch (*Perca fluviatilis*) exposed to the anxiolytic drug oxazepam show altered feeding behavior,^22^ and European starlings (*Sturnus vulgaris*) exposed to estrogenic pharmaceuticals develop longer and more attractive mating calls.^23^ There has been a recent call for experiments in behavioral ecotoxicology to reflect more realistic social complexity, that is to evaluate the effect of chemical pollutants on groups of organisms and their interactions, rather than on single organisms in isolation.^20^^,^^24^ In this respect, predator-prey interactions are an aspect of many species’ natural behavioral expression, involving multiple individuals from multiple species, that remains understudied in behavioral ecotoxicology.

Predator-prey interactions are a powerful regulator of community structure and ecosystem function,^25^ and as such, understanding how chemicals disrupt predator-prey interactions is of particular interest to ecotoxicologists. Predator-prey interactions have been shown to be affected by metal exposure. For example, mummichog (*Fundulus heteroclitus*) living in environments contaminated with metals are more likely to be captured by their predators and are slower to capture their own prey items.^26^ The outcomes of predator-prey interactions are determined by two components, each of which may be differently affected by chemical exposure: *i*) how successful predators are at capturing prey when they attack and *ii*) how successful prey are at avoiding attacks from their predators. These components determine how successful and how efficient predators are at capturing their prey. Whether exposure perturbs the performance of predator traits more than prey traits, or vice versa, is an important question with far-reaching implications for community structures and population dynamics in contaminated ecosystems.

Here, we used larval odonates, i.e., dragonfly and damselfly nymphs, to investigate the effects of two pharmaceuticals, cetirizine (a selective histamine-1 antagonist antihistamine) and citalopram (a selective serotonin reuptake inhibitor antidepressant), and their mixture on the outcomes of predator-prey interactions. Dragonfly and damselfly nymphs are an excellent model system for studying predator-prey interactions because dragonfly nymphs are voracious predators of other small aquatic organisms, including damselfly nymphs.^27^^,^^28^ Dragonfly nymphs hunt in a visually conspicuous manner—by projecting their lower jaws toward their prey in an attempt to grasp them, and this is typically associated with rapid escape swimming by damselfly prey.^27^ Damselfly nymphs also possess three leaf-shaped appendages attached to their abdomens, termed “caudal lamellae”, that can autotomize when grasped by a predator (typically when grasped by other odonates), allowing the damselfly a chance to escape.^29^^,^^30^ Autotomy is a phenomenon that has evolved across numerous taxa and is the discarding or self-amputation of a prey’s appendage when grasped by a predator. Therefore, the larval odonate study system, in which damselfly nymphs can express lamellar autotomy, allows us to conveniently quantify rates of both successful predation and unsuccessful predation attempts. Dragonfly and damselfly nymphs are also an excellent system for studying the effects of anthropogenic stressors because they show variable sensitivities to water quality and pollutants.^31^^,^^32^ We selected cetirizine and citalopram as our exposure compounds because they are commonly used and measured in freshwater environments across the globe,^6^ yet data are lacking on how they affect invertebrate behavior.

In this study, we exposed predators (dragonfly nymphs) and prey (damselfly nymphs) to either citalopram, cetirizine, and their mixture or a freshwater control and then ran two parallel predator-prey interaction experiments. In both experiments, we quantified predation success (the amount of prey that predators consumed over time), as well as predation efficiency (the number of predation attempts that resulted in a failure to capture the prey). In one experiment, predators were paired individually with prey and their behavior and activity levels were recorded over a relatively short trial period (∼1.5 h), while in the other experiment, predators were presented with groups of eight prey and were then repeatedly checked over a longer, 24 h period. We hypothesized that exposure to the pharmaceuticals would affect predation success and efficiency but remained agnostic about whether predation would become impaired or enhanced because of a lack of previous research to base our hypotheses on. This is because little to no research has been conducted to date evaluating the effects of these pharmaceuticals on the behaviors and species we focused on here. We further predicted that exposure to the mixture would have greater effects than the single exposures, perhaps due to additive or synergistic interactions.

## Results

### Experiment 1: Predation outcomes with single prey

**Figure 1:**
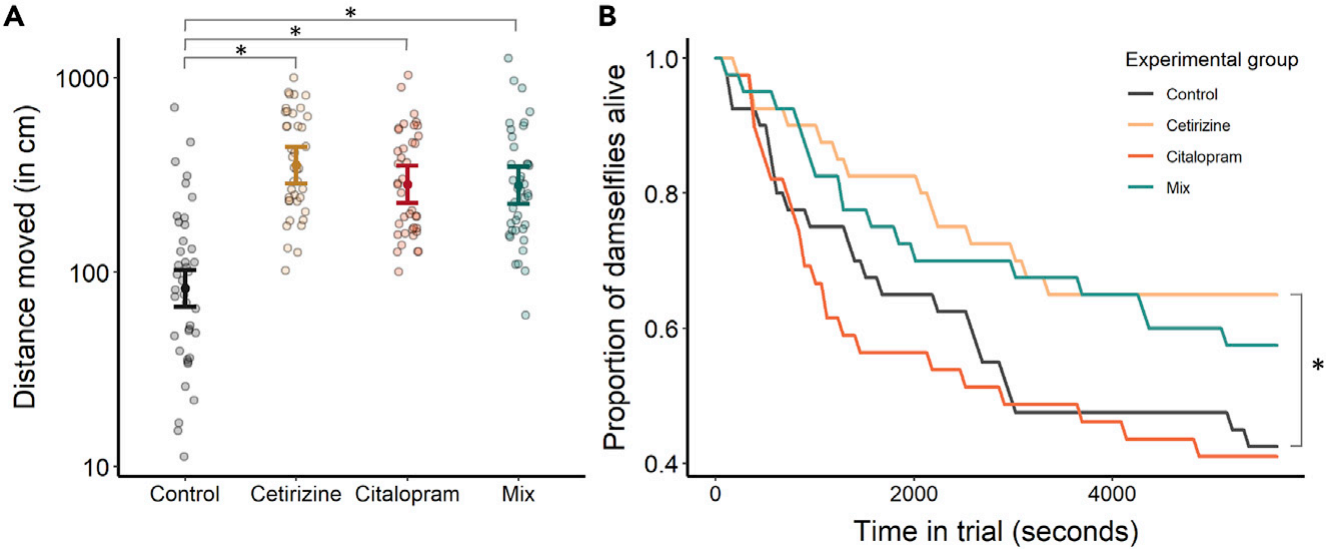
Dragonfly activity rates and predation success in Experiment 1 (A) Activity rates of dragonfly nymphs, measured as total distance moved over a 1-h period, compared across experimental groups. Model-predicted means and 95% confidence intervals for each group are overlaid on raw data points. (B) Proportion of damselfly prey still alive in each experimental group plotted against time. In both panels, ∗ indicate significant differences between groups at p < 0.05.

In the first experiment, dragonfly predators were each paired with a single damselfly prey, and both predators and prey had been exposed to the same treatment conditions as one another. All pharmaceutical treatments increased dragonfly activity (see Supplementary Materials Table S1). The average distance that each dragonfly moved in 1 h was substantially higher for all treatment groups compared to the control group (cetirizine: est. ± se = 1.46 ± 0.16, z = 9.27, p < 0.0001; citalopram: est. ± se = 1.23 ± 0.16, z = 7.76, p < 0.0001; mix: est. ± se = 1.22 ± 0.16, z = 7.69, p < 0.0001, Figure 1A). Overall, larger dragonflies also traveled farther distances (est. ± se = 0.031 ± 0.014, z = 2.27, p = 0.023).

Compared to the control group, dragonflies in the cetirizine group were significantly less successful in capturing their damselfly prey over the ∼1.5 h trial period (est. ± se = −0.93 ± 0.35, z = −2.68, p = 0.0073, Figure 1B, see Supplementary Materials Table S2). Predation success did not significantly differ between the other treatment groups and the control group (citalopram: est. ± se = 0.11 ± 0.30, z = 0.37, p = 0.71; mix: est. ± se = −0.44 ± 0.64, z = −1.38, p = 0.17). Damselflies that were larger were less likely to be predated than smaller damselflies (est. ± se = −0.16 ± 0.042, z = −3.76, p = 0.0002), and dragonflies that were larger were more likely to catch their prey than smaller dragonflies (est. ± se = 0.078 ± 0.031, z = 2.56, p = 0.010).

Over the course of their trial, the dragonflies attacked the damselflies an average (±SD) of 2.96 ± 3.23 times (range = 0–25, equating to 0.031 ± 0.034 attacks per min). Dragonflies in the control group attacked their prey an average of 3.9 times during their trials (with a success rate of 14.7%), while dragonflies in the cetirizine, citalopram, and mixture groups attacked their prey an average of 2.65 (13.2% success rate), 2.62 (22.5% success rate), and 2.65 times (16.0% success rate), respectively. The number of attack attempts made did not differ significantly between the control group and any of the treatment groups (cetirizine: est. ± se = −0.32 ± 0.22, z = −1.46, p = 0.14; citalopram: est. ± se = −0.40 ± 0.22, z = −1.82, p = 0.069; mix: est. ± se = −0.37 ± 0.22, z = −1.69, p = 0.09, see Supplementary Materials Table S3). The number of attack attempts did not differ significantly between dragonflies that were successful or unsuccessful at catching their prey by the end of the trial (est. ± se = 0.16 ± 0.16, z = 1.03, p = 0.30) or with predator size (est. ± se = −0.038 ± 0.021, z = −1.79, p = 0.074). However, attack attempts did increase as the damselfly prey got larger (est. ± se = 0.062 ± 0.027, z = −2.29, p = 0.022).

### Experiment 2: Predation outcomes with multiple prey

**Figure 2:**
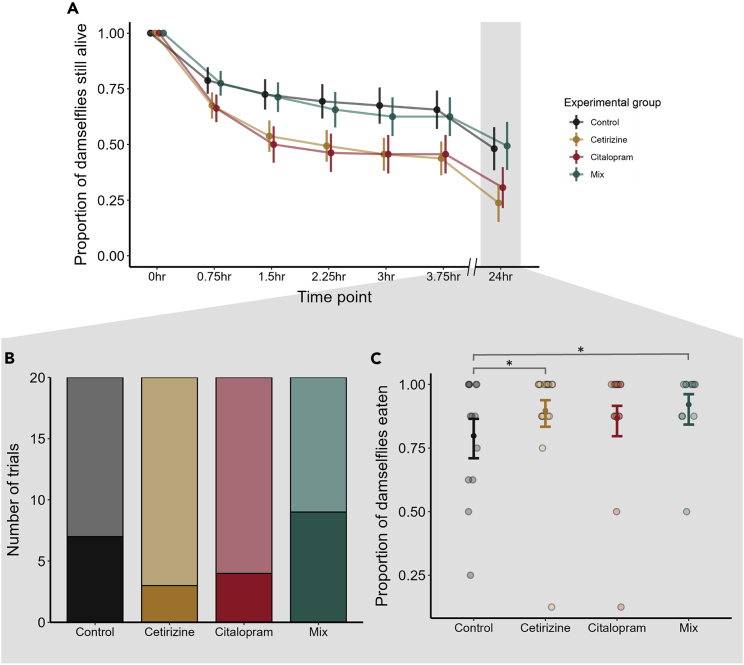
Dragonfly predation success in Experiment 2 (A) Proportion of damselfly nymphs still alive at each time point of the group survival experiment. Note the two timescales portrayed on the x axis. Dots and error bars indicate means ± standard errors. Statistical analyses of damselfly survival from Experiment 2 were performed at the 24 h time point (highlighted in gray). (B) Number of trials (out of 20 per experimental group) where the dragonfly did not consume any prey after 24 h (indicated by the darker shading). (C) The proportion of damselfly nymphs consumed per experimental group by the 24 h time point, focusing only on those trials where the dragonfly consumed prey. Dots and error bars indicate model-predicted means and 95% confidence intervals overlaid on raw data points. ∗ indicate significant differences between groups at p < 0.05.

In the second experiment, dragonfly predators were each given a group of eight damselfly prey in a larger arena than in the first experiment. Again, both the predators and prey had been exposed to the same treatment conditions as one another. Following 24 h with their prey, the dragonflies had eaten an average (±SD) of 4.15 ± 3.47 damselflies in the control group, 6.10 ± 3.06 in the cetirizine group, 5.55 ± 3.30 in the citalopram group, and 4.05 ± 3.86 in the mixture groups. In Figure 2A, we visually display how dragonfly predation success progressed up to the 24 h time point, which is the time point where we focused our analyses. At 24 h, more dragonflies in the control (n = 7/20) and mixture (n = 9/20) groups had eaten no prey at all compared to the cetirizine (n = 3/20) and citalopram (n = 4/20) groups (Figure 2B); but differences in whether or not a dragonfly ate between the treatment groups and the control group were not significant (cetirizine: est. ± se = −1.12 ± 0.78, z = −1.43, p = 0.15; citalopram: est. ± se = −0.77 ± 0.72, z = −1.05, p = 0.29; mix: est. ± se = 0.42 ± 0.65, z = 0.64, p = 0.52, see Supplementary Materials Table S4). However, of the dragonflies that did consume prey, they ate significantly more in the cetirizine and mixture groups compared to the control group (Figure 2C; cetirizine: est. ± se = 0.79 ± 0.36, z = 2.12, p = 0.034; citalopram: est. ± se = 0.50 ± 0.36, z = 1.41, p = 0.16; mix: est. ± se = 1.07 ± 0.46, z = 2.32, p = 0.021). Thus, even though the average number of damselflies eaten after 24 h was similar between the mixture and the control groups (Figure 2A), when we focus on the dragonflies that did consume prey, the mixture group and cetirizine group ate more than the controls (Figure 2C).

**Figure 3:**
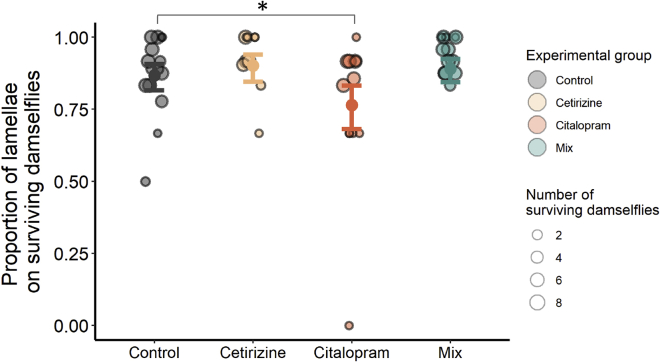
Dragonfly predation efficiency in Experiment 2 The proportion of lamellae still attached to the abdomens of surviving damselfly nymphs after 24 h, visualized by experimental group. Model-predicted means and 95% confidence intervals for each group are overlaid on the raw data points, which are scaled in size to correspond to the number of surviving damselflies. ∗ indicate significant differences between groups at p < 0.05.

We tested whether predation efficiency differed among the experimental groups over two timescales by examining the number of caudal lamellae still attached to the surviving damselflies. Over the short-term (i.e. the first 3.75 h, comprising five checks, each performed 45 min apart), the average proportion of lamellae still attached to living damselflies did not vary significantly over time (est. ± se = 0.0063 ± 0.042, z = 0.15, p = 0.88), and none of the treatment groups clearly differed from the control group (cetirizine: est. ± se = −0.27 ± 0.37, z = −0.73, p = 0.47; citalopram: est. ± se = −0.17 ± 0.37, z = −0.45, p = 0.66; mix: est. ± se = −0.14 ± 0.37, z = −0.37, p = 0.71, see Supplementary Materials Table S5). However, in the long-term after 24 h, the surviving damselflies in the citalopram exposure group had a lower proportion of their lamellae attached than those in the control group (est. ± se = −0.70 ± 0.28, z = −2.55, p = 0.011, Figure 3, see Supplementary Materials Table S6). Note that this result holds even after omitting the single lowest data point in the citalopram group (see Figure 3), which constitutes a single damselfly with zero lamellae remaining (contrast with this data point removed: est. ± se = −0.57 ± 0.25, z = −2.27, p = 0.023). This suggests that there were a higher number of failed predation attempts in this treatment group when compared to the control. The other two treatment groups did not differ significantly from the control group (cetirizine: est. ± se = 0.33 ± 0.30, z = 1.10, p = 0.27; mix: est. ± se = 0.22 ± 0.26, z = 0.84, p = 0.40).

#### Exposure concentrations and tissue uptake

The average measured concentrations of both cetirizine and citalopram in all exposure treatments, as well as the average concentrations of both compounds in the tissues of dragonflies and damselflies are reported in Table 1.

**Table 1:** Concentrations of cetirizine and citalopram measured in water samples and insect tissues

		Water (μg/L)			Tissues (ng/g)	
Experimental group		Start of exposure (Day 0)	End of exposure (Day 3)	After behavioral trials (Day 4)	Damselfly nymphs	Dragonfly nymphs
Control	[Cetirizine]	< LOQ	< LOQ	< LOQ	< LOQ	< LOQ
	[Citalopram]	< LOQ	< LOQ	< LOQ	< LOQ	< LOQ
		*N =* 6	*N =* 3	*N =* 3	*N =* 6	*N =* 17
Cetirizine	[Cetirizine]	12.86 ± 6.48	21.47 ± 2.92	6.64 ± 0.75	4.14 ± 1.22	4.04 ± 4.21
	[Citalopram]	< LOQ	< LOQ	< LOQ	< LOQ	< LOQ
		*N =* 6	*N =* 3	*N =* 3	*N =* 6	*N =* 18
Citalopram	[Cetirizine]	< LOQ	< LOQ	< LOQ	< LOQ	< LOQ
	[Citalopram]	0.60 ± 0.16	0.81 ± 0.04	0.44 ± 0.07	1.34 ± 0.33	1.76 ± 1.01
		*N =* 6	*N =* 3	*N =* 3	*N =* 6	*N =* 12
Mixture	[Cetirizine]	12.33 ± 4.76	15.21 ± 5.17	7.88 ± 0.73	3.49 ± 0.70	3.97 ± 3.33
	[Citalopram]	0.64 ± 0.24	0.81 ± 0.19	0.54 ± 0.07	1.16 ± 0.23	1.29 ± 0.82
		*N =* 6	*N =* 3	*N =* 3	*N =* 6	*N =* 18

Water was sampled from exposure tanks at the start and end of the exposure period and from the test arenas following the behavioral trials. Damselfly tissues were sampled at the end of the exposure period, and each sample required the pooling of 3–4 damselfly individuals. Dragonfly tissues were sampled from individuals after their behavioral trials.

## Discussion

We conducted two experiments investigating how two understudied pharmaceutical pollutants, citalopram and cetirizine (individually and in a mixture), affected predator-prey interactions between dragonfly nymphs and damselfly nymphs (hereafter referred to as just dragonflies and damselflies). In both experiments, we evaluated measures of dragonfly predation success (whether the predator captured any prey or how many they captured) and predation efficiency (how many failed capture attempts the predator made) but under different experimental contexts and timescales. We will therefore discuss each experiment’s results in turn before synthesizing the overall conclusions.

### Experiment 1: Predation outcomes with single prey

Dragonfly predators were each paired with a single damselfly in the first experiment. Here, we did not detect any differences in predation efficiency, as measured by the number of lunges and projections of the labium made toward the prey, between the dragonflies in the control group and those in the three pharmaceutical treatment groups. However, cetirizine-exposed dragonflies were less likely to consume their prey within the trial duration compared to the control group (i.e., predation success). Figure 1B also shows that the mixture-exposed dragonflies appeared to be less successful at consuming their damselflies (the prey survival curve for the mixture-exposed group declines less quickly relative to the control group). Taken together, this suggests that cetirizine exposure reduced the predation success of dragonflies. These results do not appear to be linked solely to differences in dragonfly activity because dragonflies in all three pharmaceutical exposure groups showed increased activity relative to the control group (discussed in the following paragraph). Despite being widely prescribed and often detected in the aquatic environment^6^^,^^33^, the impacts of cetirizine on aquatic invertebrate behavior have not been previously investigated. Previous studies have shown that exposure to cetirizine reduces metabolic capacity in clams.^34^^,^^35^ Jonsson et al.^36^ exposed damselflies to hydroxyzine or fexofenadine (two second-generation H_1_ agonist antihistamines in the same family as cetirizine) and found that the nymphs were less active. However, they also found that these two compounds bioconcentrated 100–2000 times the water concentration in damselfly tissues, which is not what we found for cetirizine in the present study. Jonson et al.^37^ also found that damselflies exposed to diphenhydramine (a first-generation H_1_ agonist antihistamine) had altered c-start escape performance. It is difficult to directly compare these previous studies with our own because of the significant differences in bioconcentration^36^ and differences in receptor selectivity between first- and second-generation antihistamines.^37^

We detected a significant increase in the activity of the dragonflies in the citalopram, cetirizine, and mixture treatments, relative to the unexposed dragonflies in the first experiment. As a selective serotonin reuptake inhibitor (SSRI) antidepressant, citalopram could interact with well-conserved serotonergic pathways that exist across vertebrates and invertebrates and may increase activity by causing behavioral disinhibition or increasing hunger or the motivation to forage.^38^^,^^39^ Comparatively less is known about histaminergic signaling and control of behavior in invertebrates even though histamine and its receptors are conserved and present in many invertebrate species.^40^ For instance, unlike in mammals, histamine plays a primary role in photoreception in many invertebrates,^41^ and therefore it is possible that the impacts of antihistamines on activity are off-target effects (i.e. not induced by their designed mode of action in humans/mammals). Regardless of mechanism, increased activity rates could have consequences for the growth-predation risk trade-off that many organisms experience in the wild, in which increased activity can permit higher success in foraging but also incurs increased susceptibility to predators.^42^

### Experiment 2: Predation outcomes with multiple prey

Dragonflies preyed upon groups of eight damselflies in a larger arena in the second experiment. Here, we observed a decrease in predation efficiency of citalopram-exposed dragonflies, as indicated by the proportion of lamellae still attached to the abdomens of surviving damselflies. This shows that citalopram-exposed dragonflies made more unsuccessful attacks on their prey, which autotomized their lamellae to escape. We did not see this pattern in the first experiment when we quantified predation efficiency via predator attack attempts. Although we can only speculate on mechanisms, it is possible that a citalopram-induced decrease in efficiency is only expressed in situations where a predator’s attention is divided among multiple prey. Citalopram is used to treat depression, panic disorders, anxiety disorders, and eating disorders^43^ and often shows disinhibitory effects (increased activity, increased boldness and exploration) in animals exposed to ecologically relevant concentrations (freshwater snails^44^; three-spine stickleback^45^^,^^46^; but see also Ziegler et al.^47^^,^^48^ and Minguez et al.^49^ for behavioral effects only at higher concentrations). The only previous study to measure the effects of citalopram (1000 ng/L) on predation success in dragonflies documented a decrease in the maximum feeding rate of dragonfly nymphs on fish fry.^50^ These results are partially consistent with our findings; while our citalopram-exposed dragonflies consumed many damselflies, they did show evidence of more failed attempts compared to controls. It is possible that our results were driven by impairment of predator hunting behavior, enhancement of prey escape behavior, or a combination of both. Future work is required to clarify any separate, and potentially asymmetric, effects on predators and prey.

When we assessed predation success, we did not detect any differences between the pharmaceutical exposures and the control group in the probability that a dragonfly would begin consuming prey. Regardless of exposure group, we noticed that an appreciable proportion of the predators had not consumed any prey after 24 h, but those that did tended to consume many prey. This was despite the fact that all dragonflies had been starved for 96–120 h prior to their behavioral trials. Of those dragonflies that did consume prey, the cetirizine- and mixture-exposed dragonflies consumed more prey than controls, while citalopram showed no statistical difference from the control (Figure 2C). Incorporating results from both experiments may help explain these results. In Experiment 1, we showed that our exposures elevated predator activity rates (Figure 1A), which could potentially facilitate their predation of damselflies through increased encounters with, and pursuit of, prey. However, citalopram exposure in Experiment 2 was also associated with a reduction in predation efficiency (Figure 3, discussed in the paragraph above). Taken together, this may explain why cetirizine- and mixture-exposed dragonflies were more successful in capturing their prey, but citalopram-exposed dragonflies were less so, when compared to controls.

### Contrasting the experiments

It is a challenge to directly compare the results of the two experiments in this study as they were conducted under different contexts (prey density, body sizes, and timescales) and measured related, but different, endpoints of predation success and efficiency. Uncovering similar findings in both experiments would have suggested to us a robust and consistent impact of any given pharmaceutical exposure on predator-prey behavior. Instead, we have found a diverging pattern of results that indicates that the impacts of these pharmaceuticals (and their mixture) on dragonfly and damselfly behavior is more nuanced. Specifically, the impacts of cetirizine on predation success in the two experiments were in opposition, indicating that the effect of cetirizine on dragonfly predation may interact with other factors. One such factor may be the predator-to-prey size ratios, which are known to impact predator behavior in *Aeshnidae* dragonfly nymphs,^51^ and the predator-to-prey size ratio was lower in Experiment 1 than in Experiment 2. However, the ratio was still not unrealistic because we found sensible effects for the impact of body size on predator-prey behavior: larger prey were more difficult for predators to capture and larger predators were better at capturing prey, indicating that our behavioral assays were well-designed and functioned as expected.

Finally, we had predicted that the mixture treatment would produce a more pronounced behavioral effect than any single pharmaceutical exposure. However, neither of our experiments revealed a clear synergistic or additive effect of the pharmaceutical mixture treatment on predator activity, efficiency, or success. Both cetirizine and citalopram have different primary modes of action via the H_1_ histamine receptor and the serotonin transporter, respectively. As such, they may have independent effects in our study species that are nonlinear in nature and require further investigation to ascertain the relative contribution of each compound to any given effect.

#### General conclusions

Overall, we revealed that short-term, sublethal exposure to the pharmaceutical pollutants cetirizine and citalopram (and their mixture) affected the predation efficiency and success of dragonfly predators on their damselfly prey. Specifically, cetirizine exposure made dragonflies less successful predators when faced with a single prey and citalopram-exposed dragonflies were less efficient predators in the group context. Furthermore, both cetirizine-exposed and mixture-exposed dragonflies were in fact more successful predators—when they started hunting—when faced with a group of prey. Both cetirizine and citalopram have been measured in surface waters on a global scale,^6^^,^^33^ yet our research is one of the first to assess the behavioral impacts of both of these compounds on dragonfly and damselfly nymphs. Our findings provide an initial indication that these two pharmaceuticals can affect aquatic insects at low concentrations and therefore open many intriguing avenues for future research. For instance, it would be beneficial to further explore the degree to which these compounds asymmetrically affect predators and prey. This is important because predator-prey outcomes are the net result of interactions between two players, whose behavior can either be impaired or be enhanced by pharmaceutical exposure. Furthermore, it would be beneficial to explore how prey sociability (e.g., group cohesion) may be pharmacologically affected, with potential consequences for their predator detection and avoidance.^20^ Additional avenues of research include assessing how exposure affects hunger and satiation in predators and quantifying a broader concentration-response curve for the behavioral effects of both compounds. Understanding the complexities of predator-prey interactions in the face of chemical pollution has important ramifications for ecosystem function in environments impacted by pharmaceutical pollution. Predator-prey dynamics underlie structure and energy flow in ecosystems, and changing such dynamics can have potentially negative consequences and/or result in new equilibria. Furthermore, pharmaceutical pollution is only expected to increase in the future due to a growing and aging human population and increased access to medications in developing markets.^52^

### Limitations of the study

A challenge of our study was the inability to directly compare both experiments (see discussion above under “comparing the experiments” for further details). It is also important to highlight that damselfly behaviors (e.g., activity and escape responses) were not tracked in this study. Thus, we cannot rule out the possibility that exposure-induced changes in damselfly behavior could have partly driven the changes in dragonfly endpoints across and within the two experiments. Lastly, although we used wild-caught animals, our experiments were conducted under controlled, yet artificial, laboratory conditions, and so future work bridging our results to the wild will be needed.

## STAR★Methods

### Key resources table

**Table:** 

REAGENT or RESOURCE	SOURCE	IDENTIFIER
**Chemicals, peptides, and recombinant proteins**		

cetirizine hydrochloride	Sigma - Aldrich	Y0001007
citalopram hydrochloride	Sigma - Aldrich	BP837
tramadol 13C, D 3	Sigma - Aldrich	T-029
Acetonitrile	Sigma - Aldrich	1000291000
Methanol	Sigma - Aldrich	1060351000
formic acid	Sigma - Aldrich	1116700250

**Deposited data**		

Raw data and R code	This paper	https://doi.org/10.17605/OSF.IO/KCF59

**Experimental models: Organisms/strains**		

*Coenagrion hastulatum*	Nydalasjön, Umeå, Sweden	N/A
*Aeshna juncae*	Nydalasjön, Umeå, Sweden	N/A

**Software and algorithms**		

R	R Core Team 2021	https://www.R-project.org/.

### Resource availability

#### Lead contact

Further information and requests for resources should be directed to and will be fulfilled by the lead contact, Aneesh P. H. Bose (aneesh.bose@slu.se).

#### Materials availability

This study did not generate new materials.

### Experimental model and subject details

#### Study species, collection, and housing

We used the nymphs of the northern damselfly (*Coenagrion hastulatum*) and the common hawker dragonfly (*Aeshna juncae*) in our study. Both species are widespread in northern and central Europe, and spend the majority of their life cycles as juveniles (i.e., nymphs) in freshwater habitats. In June 2022, we collected damselfly and dragonfly nymphs from Nydalasjön, a freshwater lake located in Umeå, Sweden (63°49′10″N, 20°20′60″E). We transported the animals to the Swedish University of Agricultural Sciences and acclimated them to temperature-controlled laboratory conditions held at 12°C for 24–48 h. During acclimation, damselflies and dragonflies were kept in separate aquaria (50 × 26 × 30 cm), at a density of approximately 100 damselflies or 20 dragonflies per 10 L of water. While in the lab, the nymphs experienced a 20 h: 4 h light: twilight cycle (60 lux: 20 lux), matching the natural light cycle they would experience in the wild given the latitude and time of year.

### Method details

#### Exposure regime

After acclimation, we exposed the damselfly and dragonfly nymphs to either cetirizine (10 μg/L), citalopram (1 μg/L), a mixture (cetirizine at 10 μg/L and citalopram at 1 μg/L; nominal concentrations), or to a freshwater control for 72 h. These experimental concentrations were chosen to match the high-end of the range of concentrations that have been detected in surface waters for these two compounds.^6^^,^^33^^,^^53^ We prepared a 2 mg/L stock solution of both cetirizine and citalopram by dissolving cetirizine hydrochloride (CAS: 83,881-52-1) or citalopram hydrochloride (CAS: 85,118-27-0) in milli-Q water. We used the stock solutions to spike the exposure tanks aiming to reach the aforementioned nominal concentrations. We monitored water quality (temperature, dissolved oxygen, pH) in the exposure tanks, which is outlined in Supplementary Materials Table S7. The nymphs were not fed during their acclimation and exposure periods. After the exposure period, we divided the nymphs into two behavioral experiments (detailed below) that were conducted over the following 24 h.

#### Sample extraction and LC-MS/MS analysis

To verify the concentrations of target compounds in water from exposure treatments and to measure their internal concentrations in experimental organisms, the samples were prepared according to the procedures described in detail previously (see McCallum et al.^54^; Cerveny et al.^55^). In short, biota samples underwent repeated solvent (acetonitrile) extraction, followed by evaporation of the supernatant, and its reconstitution (methanol), resulting in a 150 μL of final sample for analysis. Regarding the water samples, 3 mL of each sample were filtered through a 0.45 μm syringe filter and analyzed. All reported concentrations in biota samples in the present work are related to wet weight. Due to size differences between the two species, different approaches were used when processing samples for analysis. Three to four damselflies were pooled to reach the required weight for each sample, while each dragonfly nymph was homogenized, and a portion of the homogenate was extracted, as the dragonfly nymphs were too large to be analyzed whole. All samples were analyzed by liquid chromatography-tandem mass spectrometry (LC-MS/MS).

A triple stage quadrupole mass spectrometer (TSQ Quantiva, Thermo Scientific, San Jose, CA) equipped with a heated-electrospray ionisation (HESI) ion source was used for analysis of all samples. The instrument was coupled to an Accela LC pump (Thermo Fisher Scientific, San Jose, CA) and a PAL HTC autosampler (CTC Analytics AG, Zwingen, Switzerland). A C18 phase Hypersil gold column (50 × 2.1 mm ID × 3 μm particles, Thermo Fisher Scientific, San Jose, CA, USA) was used for liquid chromatography to separate the target analytes before mass spectrometry analysis. In addition to these described above, the liquid chromatography for water analysis included a Surveyor LC-Pump (Thermo Fisher Scientific, San Jose, CA, USA) and an on-line SPE Hypersil GOLD C18 column (20 × 2.1 mm ID × 12 μm particles, Thermo Fisher Scientific, Waltham, MA, USA). LC–MS-grade acetonitrile and methanol (LiChrosolv—hypergrade) were purchased from Merck (Darmstadt, Germany). Formic acid (Sigma-Aldrich, Steinheim, Germany) was used to prepare the 0.1% mobile phases for liquid chromatography. Citalopram (CAS 85118-27-0), Cetirizine (CAS 83881-52-1), and mass labeled tramadol ^13^C, D_3_ (CAS 67-56-1) were purchased at Sigma - Aldrich (Steinheim, Germany).

Linearity, precision, limit of quantification (LOQ), and measurement of blank samples were used as the quality assurance and quality control (QA/QC) for the analytical method. Quantification of target compounds was carried out using the internal standard approach. Instrumental LOQ was derived from a six-point standard curve from 0.1 to 50 ng g^−1^ and a seven-point standard curve from 0.005 to 10 μg L^−1^ for biota and water samples, respectively. Peak area corresponding to the lowest point of the calibration curve that had a signal/noise ratio of at least 10 was then used for calculation of LOQs in individual samples. Precision was expressed as a relative SD(RSD) of response factors calculated for each point of the calibration curve. Concerning the tissue samples, the analytical method was linear and precise for both citalopram (R^2^ > 0.999; RSD = 16%) and cetirizine (R^2^ > 0.999; RSD = 12%), the mean LOQs in individual samples were 0.25 and 0.23 ng g^−1^ for the two compounds. In water samples, average LOQs in individual samples were 0.01 μg L^−1^ for both citalopram and cetirizine. None of the measured drugs were found in blank samples, which were prepared at the same time as samples of experimental organisms and water samples. See Table 1 for pharmaceutical concentrations measured in the water and in insect tissues from this study.

#### Experiment 1: Predation outcomes with single prey

To test the impacts of cetirizine, citalopram and their mixture on predator activity and how predators interacted with their prey, we placed a single damselfly nymph into Petri dish arenas (9 cm diameter, 50 mL water) and gave them a 5-min acclimation period. Damselfly nymphs use lamellae, leaf-shaped appendages attached to their abdomens, to propel themselves while swimming and can also autotomize them to escape the grasp of predators.^56^ Therefore, we only used damselfly nymphs with at least two lamellae attached to their abdomens to ensure that no damselflies were significantly impaired in their swimming speed and escape capacity.^56^ We then added a dragonfly nymph to the dish with them, and we filmed the pair from above (using a GoPro Hero 8 Black video camera) for 5656 s. This time frame was based on the duration of eight video segments generated by GoPro cameras while filming, and corresponds to ∼94 min. The body sizes of the nymphs were measured from still-frames extracted from the videos in ImageJ. Average body size of the dragonflies (average ±SD = 2.16 ± 0.42 cm, ANOVA: F_3,76_ = 0.67, p = 0.57) and the damselflies (average ±SD = 1.39 ± 0.30 cm, ANOVA: F_3,155_ = 1.39, p = 0.25) did not differ across experimental groups. Only nymphs that were exposed to the same pharmaceutical treatment were placed in pairs together, and each Petri dish contained water containing with the same treatment as the nymphs had experienced during their 72 h exposures (see Table 1). If after 6 h, the dragonfly had not eaten their damselfly, the damselfly was removed and not used again in this study. The following day, we repeated the trials by presenting the dragonfly with a new damselfly, and we filmed their interactions for another 5656 s. Ten experimental replicates were conducted per treatment and this experiment was also repeated a second time one week apart, amounting to n = 20 replicates per treatment and a total of 80 dragonflies and 160 damselflies used (although in one trial, the dragonfly escaped its Petri dish and was therefore omitted).

The whole videos were scored by an observer who was blind to treatment. They scored the number of attack attempts made by the dragonfly toward the damselfly. Attack attempts were counted each time the dragonfly approached the damselfly resulting in the damselfly attempting to swim away rapidly (attack attempts would often involve the dragonfly projecting its lower jaw, i.e., labium, toward the damselfly). We also quantified activity levels of the dragonfly, measured as total distance moved (cm), over the first hour once both nymphs were in the dish. Note that only the dragonfly movement was quantified in these trials, as the smaller damselfly larvae could not be confidently tracked given our experimental setup. We tracked activity using the automated computer software EthoVision XT (Noldus Information Technology).

#### Experiment 2: Predation outcomes with multiple prey

To test the impacts of cetirizine, citalopram and their mixture, on the predation success of dragonflies on damselflies in a group context, we placed groups of eight damselflies in test aquaria (12 × 20 cm, water depth 2.1 cm, 500 mL water). We gave the damselflies a 15-min acclimation period before a dragonfly predator was added. We again used only damselflies that had at least two lamellae, and the experimental groups did not differ from one another in the proportion of lamellae attached to the damselfly abdomens at the start of the trials (control: damselflies started the trials with an average of 94.8% of their lamellae; cetirizine: 93.8%; citalopram: 94.2%; mixture: 95%). We took a top-down photograph of each test aquarium after the dragonfly was added to measure body sizes (ImageJ, version 1.53k): the average body sizes of the dragonflies (average ± std. dev. = 3.59 ± 0.32 cm, ANOVA: F_3,76_ = 0.58, p = 0.63) and the damselflies (average ± std. dev. = 1.00 ± 0.11 cm, ANOVA: F_3,76_ = 1.89, p = 0.14) did not differ across experimental groups. Only insects that were exposed to the same pharmaceutical treatment were placed in groups together, and each test aquarium contained water that contained the same treatment as the nymphs had experienced during their 72 h exposures. Ten replicate groups were formed per exposure treatment, and this experiment was repeated twice, one week apart, amounting to n = 20 groups per treatment and a total of 640 damselflies and 80 dragonflies used. We conducted ‘spot checks’ to count the number of lamellae remaining on the surviving damselflies’ abdomens. We checked on the groups every 45 min following group formation, five times (therefore over ∼3 h 45 m), and then a sixth and final check was performed 24 h after group formation.

### Statistical analyses

#### Experiment 1: Predation outcomes with single prey

We compared dragonfly activity rates (i.e. distance moved; log-transformed) among the treatment groups using a linear mixed effects model (LMM; using the ‘glmmTMB’ R package^57^). Treatment group and predator size were included as predictor variables, and dragonfly ID was included as a random intercept.

To compare the rates of dragonfly prey capture among treatment groups, we used a Cox proportional hazards mixed-effects survival model (using the ‘coxme’ R package^58^). Treatment group (4-level categorical variable), predator size (in mm), and prey size (in mm) were included as predictor variables. We also included a random intercept of dragonfly ID to account for the non-independence among data points arising from presenting two damselfly prey to each dragonfly one day apart.

Next, we tested whether the number of attacks attempted by each dragonfly toward their prey differed among treatment groups, and we fit a generalised linear mixed-effects model (GLMM) assuming a negative binomial error distribution (using ‘nbinom2’ family in ‘glmmTMB’ R package). Specifically, the number of attack attempts made by the dragonfly was modeled as the response variable. We included treatment group, predator size, prey size, and a 2-level categorical variable indicating whether the dragonfly was successful at catching their prey within the allotted trial period as predictor variables. As above, a random intercept of dragonfly ID was included.

#### Experiment 2: Predation outcomes with multiple prey

We fit a zero-inflated, generalized linear model (using the ‘glmmTMB’ R package) assuming a binomial error distribution to the proportion of damselfly prey that were consumed by the dragonfly predators after 24 h. We included treatment group in both the conditional and zero-inflated model components. Thus, this model examined whether treatment groups differed in the probability that predators would eat any prey at all (versus no prey), and then also examined, among the predators that did consume prey, whether treatment groups differed in the average proportion of damselflies eaten.

Next, we examined whether the treatment groups differed in how many attacks the prey had avoided by examining the number of lamellae still attached to the damselfly abdomens. We fit two GLMMs assuming a beta error distribution for proportion data (using the ‘glmmTMB’ R package). One model focused on short-term patterns occurring over the first five time points, spanning 3.75 h, and the second model focused on long-term patterns at the 24 h time point. The response variable was the number of lamellae still attached to the abdomens of the living damselflies expressed as a proportion (using the Smithson and Verkuilen^59^ transformation) of the total possible lamellae that they could have had (each damselfly has maximally three lamellae). Individual data points were weighted by the square root of the number of surviving damselflies at each time point. Both models included treatment as a predictor variable, and the short-term model also included time point as a continuous covariate. For the short-term model, we also fitted random slopes of group ID over time.

#### General statistical methods

All of the above analyses were conducted using the statistical computing program R (v. 4.1.2^60^). In all statistical tests, we limited the number of pairwise comparisons by only comparing each pharmaceutical treatment (cetirizine, citalopram, or mix) to the control group. Interactions among predictor variables were tested for, but dropped from the final models if they did not significantly improve model fit based on a likelihood ratio test.

## Data Availability

All original data and code have been deposited with The Open Science Framework and are publicly available as of the date of publication. DOIs are listed in the key resources table. Any additional information required to reanalyze the data reported in this paper is available from the lead contact upon request.
