# Supplementary material for: Pharmaceutical pollution disrupts the behavior and predator-prey interactions of two widespread aquatic insects
Source: iScience. 2022 Nov 25;25(12):105672. doi: 10.1016/j.isci.2022.105672 (PMC9758520; doi:10.1016/j.isci.2022.105672)
Supplement: Document S1. Tables S1–S — 7 [file mmc1.pdf]

iScience, Volume *25*

Supplemental information

Pharmaceutical pollution disrupts the behavior and predator-prey interactions of two widespread aquatic insects

Aneesh P.H. Bose, Erin S. McCallum, Mladen Avramović, Michael G. Bertram, Eva-Lotta Blom, Daniel Cerveny, Sara N. Grønlund, Johan Leander, Petter Lundberg, Jake M. Martin, Marcus Michelangeli, Lo Persson, and Tomas Brodin

SUPPLEMENTARY MATERIALS

Supplementary Materials Table S1: Results of linear mixed effects model examining activity rates of the dragonfly nymph predator in Experiment 1 as measured by total distance moved (log-transformed). The control group is used as the reference level. Estimates are given in terms of the difference in the expected (log) response variable for each unit increase in a continuous predictor, or between groups for a categorical predictor. Related to Figure 1A.

**Term**	**Estimate ± SE**	**z-value**	***P***
Intercept	3.74 ± 0.32	11.67	**< 0.0001**
Cetirizine	1.46 ± 0.16	9.27	**< 0.0001**
Citalopram	1.23 ± 0.16	7.76	**< 0.0001**
Mix	1.22 ± 0.16	7.69	**< 0.0001**
Predator body size (mm)	0.031 ± 0.014	2.27	**0.023**

Supplementary Materials Table S2: Results of Cox proportional hazards mixed effects model examining survival rates of damselfly prey in Experiment 1. The control group is used as the reference level. The exponential of the estimate can be interpreted as the hazard ratio for each unit increase in a continuous predictor, or between groups for a categorical predictor. Related to Figure 1B.

**Term**	**Estimate ± SE**	**exp(Estimate)**	**z-value**	***P***
Cetirizine	-0.93 ± 0.35	0.40	-2.68	**0.0073**
Citalopram	0.11 ± 0.30	1.11	0.36	0.72
Mix	-0.44 ± 0.32	0.64	-1.38	0.17
Predator body size (mm)	0.078 ± 0.031	1.08	2.56	**0.01**
Prey body size (mm)	-0.16 ± 0.042	0.85	-3.76	**0.0002**

Supplementary Materials Table S3: Results of negative binomial generalized linear mixed effects model examining the number of predation attempts made by the dragonfly predators in Experiment 1. The control group is used as the reference level. Estimates are given in terms of the difference in logs of the expected counts of the response variable for each unit increase in a continuous predictor, or between groups for a categorical predictor. Related to STAR Methods.

**Term**	**Estimate ± SE**	**z-value**	***P***
Intercept	1.17 ± 0.49	2.36	**0.018**
Cetirizine	-0.32 ± 0.22	-1.46	0.14
Citalopram	-0.40 ± 0.22	-1.82	0.069
Mix	-0.37 ± 0.22	-1.69	0.09
Predator body size (mm)	-0.038 ± 0.021	-1.79	0.074
Prey body size (mm)	0.062 ± 0.027	2.29	**0.022**
Success at catching prey (yes/no)	0.16 ± 0.16	1.03	0.30

Supplementary Materials Table S4: Results of zero-inflated binomial generalized linear mixed effects model examining predation success of dragonfly nymphs in Experiment 2. The control group is used as the reference level. The estimates from the conditional model are given in terms of the log of the odds ratio for successful predation events occurring between groups. The estimates from the zero-inflated model are given in terms of the log of the odds ratio for the non-occurrence of any successful predation event occurring between groups. Related to Figure 2B & C.

**Term (zero-inflated model)**	**Estimate ± SE**	**z-value**	***P***
Intercept	-0.62 ± 0.47	-1.32	0.19
Cetirizine	-1.12 ± 0.78	-1.43	0.15
Citalopram	-0.77 ± 0.73	-1.05	0.29
Mix	0.42 ± 0.65	0.64	0.52

**Term (conditional model)**	**Estimate ± SE**	**z-value**	***P***
Intercept	1.37 ± 0.24	5.63	**< 0.0001**
Cetirizine	0.79 ± 0.37	2.12	**0.034**
Citalopram	0.50 ± 0.36	1.41	0.16
Mix	1.07 ± 0.46	2.32	**0.021**

Supplementary Materials Table S5: Results of beta generalized linear mixed effects model examining the proportion of lamellae (transformed following Smithson and Verkuilen 2006) still attached to damselfly prey across the first 3.75 hrs of Experiment 2. The control group is used as the reference level. Estimates are given in terms of the log of the odds ratio for a lamella to be autotomized for each unit increase in a continuous predictor, or between groups for a categorical predictor. Related to STAR Methods.

**Term**	**Estimate ± SE**	**z-value**	***P***
Intercept	3.26 ± 0.26	12.42	**< 0.0001**
Time point (continuous variable from 0 – 5)	0.0063 ± 0.042	0.15	0.88
Cetirizine	-0.27 ± 0.37	-0.73	0.47
Citalopram	-0.17 ± 0.37	-0.45	0.66
Mix	-0.14 ± 0.37	-0.37	0.71

Supplementary Materials Table S6: Results of beta generalized linear mixed effects model examining the proportion of lamellae (transformed following Smithson and Verkuilen 2006) still attached to damselfly prey after 24hrs in Experiment 2. The control group is used as the reference level. Estimates are given in terms of the log of the odds ratio for a lamella to be autotomized between groups. Related to Figure 3.

**Term**	**Estimate ± SE**	**z-value**	***P***
Intercept	1.88 ± 0.20	9.42	**< 0.0001**
Cetirizine	0.33 ± 0.30	1.097	0.27
Citalopram	-0.70 ± 0.28	-2.55	**0.011**
Mix	0.22 ± 0.26	0.84	0.40

Supplementary Materials Table S7: Water quality parameters measured from exposure tanks (average ± std. dev.). Related to STAR Methods.

Experimental group	Dissolved oxygen (%)	Dissolved oxygen (mg/L)	Temperature (°C)	pH
Control	88.05 ± 10.65<br>*N* = 6	9.36 ± 1.13<br>*N* = 6	12.47 ± 0.21<br>*N* = 6	7.57 ± 0.06<br>*N* = 3
Cetirizine	88.90 ± 10.77<br>*N* = 6	9.47 ± 1.13<br>*N* = 6	12.52 ± 0.22<br>*N* = 6	7.66 ± 0.06<br>*N* = 3
Citalopram	89.52 ± 10.39<br>*N* = 6	9.64 ± 1.07<br>*N* = 6	12.38 ± 0.22<br>*N* = 6	7.61 ± 0.03<br>*N* = 3
Mix	88.33 ± 10.44<br>*N* = 6	9.45 ± 1.09<br>*N* = 6	12.35 ± 0.18<br>*N* = 6	7.67 ± 0.03<br>*N* = 3
